# Pilot study of minimally adherent silver dressings for acute surgical wounds

**DOI:** 10.1002/hsr2.865

**Published:** 2022-10-03

**Authors:** Meredyth B. Berard, Frank H. Lau

**Affiliations:** ^1^ School of Medicine Louisiana State University Health Sciences Center New Orleans Louisiana USA; ^2^ Division of Plastic and Reconstructive Surgery, Department of Surgery Louisiana State University Health Sciences Center New Orleans Louisiana USA

**Keywords:** acute surgical wounds, Mepitel®, minimally adherent silver dressing, prospective randomized controlled trial

## Abstract

**Background and Aims:**

Minimally adherent silver dressings (SILVER MASD) are antimicrobial, nonirritating, provide a moist wound healing environment, and low cost. The purpose of this pilot, single‐center, non‐blinded randomized controlled trial was to quantify the outcomes of acute surgical wounds treated with MASD versus standard of care (SoC) dressings.

**Methods:**

Thirty‐two patients with acute wounds were randomized 1:1 to be treated with MASD once weekly or SoC following surgical excision of skin and/or subcutaneous tissue between September 13, 2016 and November 28, 2017. The outcome variables included clinical infection, time to wound closure, and pain scores at dressing changes. Two independent, one‐sided sample *t*‐tests were performed to assess statistical significance.

**Results:**

There was no difference in wound healing between SILVER MASD and SoC. Dressing changes were less painful for wounds managed with MASD silver dressings.

**Conclusions:**

The results of this study suggest that MASD are not less effective in wound healing compared to SoC while also providing the benefit of decreased pain at dressing changes. Therefore, minimally adherent silver dressings can and should be considered a viable option in the management of acute surgical wounds.

## 1 INTRODUCTION

Wound care remains a major health care burden. In the United States alone, wounds cost approximately $25 billion per year.^1^, ^2^ Reducing this cost burden requires low‐cost, new technologies that meet the requirements of “ideal” wound dressings: minimally adherent to reduce dressing change pain and preserving of a moist healing environment without allowing fluid accumulation.

Silicone minimally adherent dressings (Mepitel®, Mölnlycke Health Care, US, LLC) are primary wound contact dressings that provide gentle adhesion, are nonirritating, and when paired with a secondary dressing layer, can effectively manage fluid accumulation. The permeable nature of silicone preserves a moist wound‐healing environment that is complemented by the absorptivity of a secondary dressing. Fenestrations within the dressing avoid the accumulation of fluid that can predispose to infection which makes it possible to change only an absorbent secondary dressing layer, leaving the silicone dressing in place. It is low cost, at $0.25 per square cm, or about $19.00 for the smallest size dressing, requires no durable medical equipment, and can be left unchanged for up to 14 consecutive days.^3^ A pilot, single‐center, non‐blinded randomized controlled trial (RCT) found that patients whose skin‐graft donor sites were dressed with silicone dressings demonstrated the fastest healing time and least duration of pain when compared to traditional moist‐to‐dry dressings.^4^

With the growing recognition that more than 80% of bacterial infections are associated with biofilms,^5^ antimicrobial and anti‐biofilm activity is now included in the definition of the ideal wound dressing.^6^ In modern wound dressings, several antimicrobial substances are used including iodine, honey, and ionic silver. Ionic silver has a broad spectrum of antimicrobial activity, most of which is facilitated by silver ions' ability to react with bacteria membrane proteins and DNA, disrupting DNA replication and denaturing proteins.^7^ Moreover, ionic silver confers additional wound‐healing benefits including antiplatelet activity, antioxidant effects, immunity enhancement, wound healing and bone regeneration, and an increase in antibiotic efficiency.^8^ Bioactive silver also damages bacterial cell walls and dismantles pathogens' electron transport system, and it perforates cytoplasmic membranes, which induces metabolite loss and cell death.^7^

Silicone minimally adherent dressings (Mepitel® AG) are approved for sale in the United States.^9^ The purpose of this study is to establish the outcomes of acute surgical wounds treated with MASD compared to standard of care (SoC) dressings through a pilot, prospective RCT.

## 2 MATERIALS AND METHODS

An IRB‐approved, pilot, prospective RCT was performed on patients undergoing surgical excision of skin and/or subcutaneous tissue between September 13, 2016 and November 28, 2017. Informed consent was obtained from all subjects. Subjects were randomized 1:1 to have sites dressed with either silver MASD changed weekly, or SoC. Patients receiving silver MASD used ABD pads (Medline) as secondary dressings; the ABD pads were secured with paper tape and changed daily or more frequently as needed for saturation. For superficial wounds, SoC was Xeroform™ Occlusive Petrolatum Gauze (Medtronic) dressings. For deep wounds, SoC was moist‐to‐dry gauze dressings changed twice a day. Wounds were classified as superficial if only partial to full thickness skin excision was performed and deep if all layers of skin and underlying subcutaneous tissue were involved. Primary endpoints included: (1) frequency of clinical infection, (2) time to wound closure, and (3) pain scores at dressing changes. Pain scores were measured by patient‐reported visual analog scale rating from 0 (no pain) to 10 (maximum pain). Wounds were assessed weekly. Data were stored in a HIPAA‐compliant, firewall‐protected REDCap database. Two independent, one‐sided sample *t*‐tests were performed to analyze differences of statistical significance (*p* < 0.05) between silver MASD and SoC using GraphPad Prism 7 (GraphPad Software, Inc.).

## 3 RESULTS

Thirty‐two patients were enrolled in the trial and randomized 1:1 to silver MASD versus SoC. Six subjects withdrew without undergoing any surgical procedures and one patient did not complete their follow‐up visits. Of the 25 patients who had acute wounds, demographics (Table 1) included: 16 males (64% or 16/25), 9 females (36% or 9/25), average age of 45.6 years (SD 18.4 years), and average body mass index of 29.7 (SD, 10.5). Race included 15 African American (60.0% or 15/25), 8 white (32.0% or 8/25), 1 Hispanic (4.0% 0r 1/25), and 1 Asian (4.0% or 1/25) patient.

**Table 1:** Patient demographics

Gender	
Male	16 (64.0%)
Female	9 (36.0%)
Age in years	45.6 (SD 18.4)
BMI	29.7 (SD 10.5)
Race	
African American	15 (60.0%)
Caucasian	8 (32.0%)
Hispanic	1 (4.0%)
Asian	1 (4.0%)

Abbreviation: BMI, body mass index.

**Figure 1:**
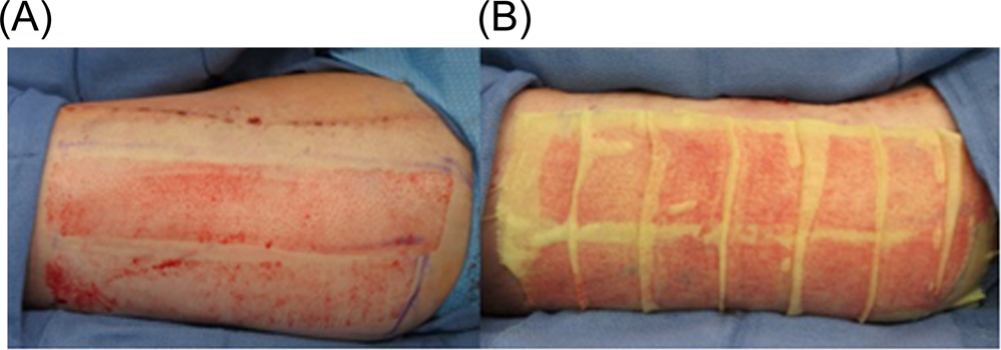
Superficial acute surgical wound (A) with Xeroform dressing (B)

**Figure 2:**
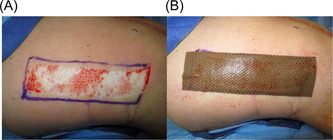
Superficial acute surgical wound (A) with Mepitel® Ag dressing (B)

The average length of follow‐up was 5.7 weeks (SD, 3.8). Ten patients were randomized to SoC (Figure 1) and 15 were randomized to silver MASD (Figure 2). There was no statistical difference in the average initial wound size (SoC 161.2 cm^2^ vs. silver MASD 136.9 cm^2^, *p* = 0.71). With regard to the primary endpoints of this study (Table 2), there was no significant difference in infection rates, with 1 infection (10.0% or 1/10) in the SoC arm and 1 (6.7% or 1/15) in the silver MASD arm. There was no significant difference in time to wound closure (SoC 6.0 vs. silver MASD 4.4 weeks, *p* = 0.15). Wounds that healed via secondary intention also had similar average healing rates (SoC 64.1% vs. silver MASD 65.7%, *p* = 0.91).

**Table 2:** Wound characteristics and results during follow‐up

	MASD	SoC	*p*
No. of subjects	15	10	
Deep wounds	4	1	
Superficial wounds	11	9	
Avg. initial wound size (cm^2^)	136.9	161.2	0.710
Avg. healing rate	65.7%	64.1%	0.911
Avg. time to closure (wks)	4.4	6.0	0.151
Avg. visual analog pain score	1.1	2.8	**0.008**
Infection rate	6.7%	10.0%	

Abbreviation: Avg., average; MASD, minimally adherent silver dressing; No., number; SoC, standard of care; wks, weeks.

Notably, wounds treated with silver MASD were significantly less painful (SoC 2.8 pain score vs. MASD 1.1, *p* = 0.008).

## 4 DISCUSSION

As the incidence and cost burden of wounds continues to rise, the search for an ideal wound dressing is ever more pressing. The results of our pilot, prospective, randomized controlled trial suggest that there was no statistical difference in time to wound closure or healing rates, suggesting that silver MASD are not less effective in healing wounds than SoC. Additionally, the silver MASD reduced patient pain, the frequency of dressing changes, and the cost of wound care, making it a reasonable alternative to SoC.

Previous literature demonstrated that another MASD (Mepilex® Ag) offers value in the management of wounds.^10^ In an RCT comparing Mepilex Ag to silver sulfadiazine application in patients with partial thickness burns, wounds managed with Mepilex Ag healed faster, were less painful at dressing changes, had significantly lower mean daily hospital charges, and lower costs for dressing changes and narcotics.^10^ Mepitel Ag contains the same antimicrobial silver compound and proprietary silicone‐based minimally adherent as the dressing used in the RCT. Its design goes a step further by allowing it to remain in place while secondary, absorptive dressings are changed, as needed. This may enable even easier and faster‐dressing changes. Due to the small sample size of deep wounds in this particular study, it is difficult to definitively conclude a difference in infection prevention between silver MASD and SoC, and thus the antimicrobial benefit of silver MASD. However, it is worth noting that the only deep wound dressed with SoC developed infection (100%), whereas only one of four dressed with silver MASD developed clinical signs of infection (25%).

Our data suggest that silver MASD is a reasonable option for management of acute surgical wounds due to its non‐occlusive, minimally adherent nature, and its innate soft, pliable properties. These characteristics make silver MASD appropriate for surgical wounds. Silver ions reduce the microbial load in the dressing and may contribute to healing without bacterial colonization.^11^, ^12^, ^13^ The silver MASD's design and structure allow for wound exudate to pass through the dressing and retain a desirable level of wound moisture. Conformability enables this dressing to be effectively used in wounds located in anatomically difficult areas such as the groin, axilla, and perineum. These qualities can also contribute to a longer wear time. In wound management, utilizing an advanced dressing with extended wear time can reduce both hard and soft costs. One example included a 77‐year‐old female who was admitted into critical care for sepsis, right foot cellulitis, urinary tract infection, and toxic epidermal necrolysis. By comparing SoC with a similar silver MASD, a total cost savings of $3769.38 (Table 3) was realized by the hospital.

**Table 3:** Schindler P; Integument Emergency: A case study of toxic epidermal necrolysis; Presented at WOCN 2016 Comparison of Two Wound Therapies

Product	Topical emollient and oil emulsion mesh	Absorbent soft silicone antimicrobial exudate transfer foam dressing with a superabsorbent cover – utilized × 12 days
Pain at dressing changes	High	None
Pain medication	Dilaudid before each dressing (4× daily and PRN breakthrough pain)	None
Patient satisfaction	Poor	High
Exudate management	Poor – as evidenced by peri‐wound maceration and strike‐through drainage	Good – a conformable foam contact with a superabsorbent cover dressing allows drainage to move vertically into the secondary absorbent dressing thus controlling large volumes of exudate while providing a moist wound environment
Antimicrobial protection	None	Sustained effect up to 14 days
Number of dressing changes ordered over 12 days	48 minimum At least 4× daily and PRN with strike‐through	2 over 12 days Also visually assessed the contact layer daily
Nursing time for dressing change/cost of labor	1.5 h × 48 = 72 h total Labor costs: $2484.00 (72 h × $34.50^6^)	1.5 h × 2 = 3 h total Labor costs: $103.50 (3 h × $34.50^6^)
Nursing perception regarding ease of application	Difficult	Easy
Dressing, pain medication, and labor cost estimates	$4072.32	$302.94

*Note*: Total cost savings (supplies/pain medications/labor) – $3769.38.

Silver has been shown to decimate bacterial populations including methicillin‐resistant *Staphylococcus epidermidis*, vancomycin‐resistant *Enterococcus*, extended spectrum B‐lactamase producing *Klebsiella*, *Pseudomonas aeruginosa*, ampicillin‐resistant *Escherichia coli* O157:H7, and erythromycin‐resistant *Streptococcus pyogenes*. Fungicidal activity against *Candida albicans* and *Candida glabrata*, which are difficult to treat because of formation of biofilms resistant to prescription antifungals, has also been noted.

The combined benefits of non‐occlusive silicone dressings with antimicrobial properties of silver allow for a decrease in the frequency of dressing changes and associated costs, as well as improved overall healing in terms of decreased risk of infection, pain, and skin trauma induced during dressing changes.^14^ Our results show similar pain scores at dressing changes in all wounds, however, only one patient with a deep wound was randomized to SoC, while five patients with deep wounds were randomized to silver MASD group, which could explain the perceived lack of difference between the groups. Of note, SoC dressings for deep wounds included twice a day dressing changes, while silver MASD group only required weekly dressing changes, supplementing the decreased cost.

A limitation of this study was that attaining a pain score at each dressing change for the deep wound in SoC group was not feasible. For this reason, we also assessed differences in pain scores between the two groups with superficial wounds. This showed that average pain scores at time of dressing changes in superficial surgical wounds were significantly lower in the silver MASD group.

This study demonstrates the advantages of silver MASD for acute surgical wounds. This trial confirms published results from prior studies in which silver and non‐occlusive dressing regimens were individually demonstrated to successfully manage wounds, and at the same time, introduces the concept of optimization in wound management by harnessing the benefits of both modalities in a singular dressing. Silver MASD possess additional qualities, such as a decrease in pain at dressing changes, overall cost, and frequency of necessary changes, as well as noted ease in application, that display at the very least that silver MASD are a reasonable alternative to SoC. In accordance with our findings, silver MASD (Mepitel® Ag), through a multifactorial mechanism of wound management, should be considered in the management of acute surgical wounds.

## AUTHOR CONTRIBUTIONS

**Meredyth B. Berard**: Writing – review and editing. **Frank H. Lau**: Conceptualization; Formal analysis; Funding acquisition; Investigation; Methodology.

## CONFLICT OF INTEREST

The authors declare no conflict of interest.

## TRANSPARENCY STATEMENT

The lead author Frank H. Lau affirms that this manuscript is an honest, accurate, and transparent account of the study being reported; that no important aspects of the study have been omitted; and that any discrepancies from the study as planned (and, if relevant, registered) have been explained.

## Data Availability

The authors confirm that the data supporting the findings of this study are available within the article.
